# Seroprevalence of hepatitis B and C virus infections among diabetic patients in Kisangani (North-eastern Democratic Republic of Congo)

**DOI:** 10.11604/pamj.2018.31.160.17176

**Published:** 2018-11-02

**Authors:** Paul Kambale Kombi, Salomon Batina Agasa, Jean Paulin Mbo Mukonkole, Lucien Bolukaoto Bome, Camille Atoba Bokele, Charles Kayembe Tshilumba

**Affiliations:** 1 Département de Médecine Interne, Cliniques Universitaires de Kisangani, Faculté de Médecine et de Pharmacie, Université de Kisangani, République Démocratique du Congo

**Keywords:** Hepatitis B virus, hepatitis C virus, diabetes mellitus, seroprevalence

## Abstract

**Introduction:**

The link between diabetes mellitus and hepatitis B and C Virus infections has not yet been studied in the Democratic Republic of Congo, a country where diabetes mellitus is a growing disease and the prevalence of hepatitis B and C viruses infections is high. The aim of this study was to determine the seroprevalence of these viruses in diabetic patients.

**Methods:**

We conducted a descriptive cross-sectional study in diabetic subjects attending Kisangani University Clinics and General Hospitals of Kisangani City as well as the Diabetics Association of Oriental Province. The control group consisted of volunteer blood donors recruited from the Kisangani Provincial Blood Transfusion Center. Blood glucose was measured with the spectrophotometer; for hepatitis B and hepatitis C viruses serology, we used rapid test kits (Determine TM^®^ HBsAg and Hexagon^®^ HCV test) and ELISA if seropositivity by rapid tests. The analysis was done by SPSS software.

**Results:**

Seroprevalence of hepatitis C virus in diabetics was 24.8% compared to 1.9% in volunteer blood donors (p = 0.0000); that of hepatitis B virus was 3.4% versus 3.5% in volunteer blood donors (p = 0.906). Hepatitis C virus infection was more common in type 2 diabetics (p = 0.006) and significantly associated with age of diabetic patients (p = 0.002).

**Conclusion:**

The seroprevalence of hepatitis C virus and not hepatitis B virus infection is significantly high in diabetic subjects, particularly type 2 diabetics, in the Democratic Republic of Congo and suggests systematic screening for this infection in any diabetic patient.

## Introduction

Diabetes mellitus and viral hepatitis B and C are serious public health problems affecting respectively 387 million, 350 million and 170 million people worldwide [1-3]. About 90% of people infected with the hepatitis B virus (HBV) evolve spontaneously towards healing, but 80-85% of those infected with hepatitis C virus (HCV) become chronic carriers with a risk of developing cirrhosis and hepatocellular carcinoma [4]. This risk is particularly high and the progression more rapid when the infection occurs in a diabetic subject [5]. For almost two decades, the link between diabetes mellitus and viral hepatitis B and C has been the subject of intense works around the world. This link was assessed either by measuring the prevalence of HBV and C markers in the diabetic patients population, or by looking for glucose intolerance and / or diabetes mellitus in those infected with these viruses. Regarding the seroprevalence of these viruses in diabetics, the results remain conflicting. Some studies have reported high seroprevalence of HBV and HCV among diabetics [2, 6-8] while others have not observed a significant difference compared to control groups [9-11]. In Kisangani, a city in the north-eastern of the Democratic Republic of Congo, diabetes mellitus is a growing disease [12, 13]. In addition, seroprevalence of HBV and C in blood donors was estimated at 5.4% and 4.1%, respectively [14, 15]. However, at our knowledge, the association between diabetes mellitus and viral hepatitis B and C has not yet been studied in the Democratic Republic of Congo. The knowledge of this association will contribute to the improvement of the management of diabetes mellitus and the monitoring of the evolution of viral hepatitis B and C. The aim of this study was to determine the prevalence of serological markers of HBV and C in diabetic subjects in the Democratic Republic of Congo.

## Methods

From April 15, 2013 to march 31, 2015, we conducted a descriptive cross-sectional study in diabetic subjects as well as volunteer blood donors in Kisangani. For diabetics, these were patients attending Kisangani University Clinics as well as general hospitals in Kisangani City. Other patients were recruited from the Diabetics Association named **« Association des Diabétiques de la Province Orientale »** during the same period. Voluntary blood donors were recruited at the Kisangani Provincial Blood Transfusion Center. The sample size of diabetics was determined based on the seroprevalence of HBV 6.3% [16] using the formula

$$n=\frac{\left({\text{z}}^{2}*p*1-p\right)}{{\text{d}}^{2}}$$

where p = the desired degree of precision of 95%. It gave us 90,672. But, anticipating the non-respondents, we added a proportion of 10%, bringing the total number to about 100. In definitive, 149 subjects were enrolled in the study, including 63 women and 86 men. The mean age was 54 ± 13 years with limits of 15 and 83 years. Included in the study were previously known diabetic subjects attending or not attending the above-mentioned health facilities for diabetes mellitus, and who agreed to participate in the research. Subjects with gestational diabetes, diabetics under the age of 15 years, and subjects with hyperglycemia following long-term corticosteroid therapy were excluded from the study. About volunteer blood donors, we enrolled 5259 subjects, all of whom donated during the study period. Of these subjects, 138 (2.62%) were female and 5121 (97.38%) were male. Their mean age was 24.64 ± 7.77 years. Diabetes mellitus was defined according to the criteria of the American Diabetes Association (ADA) of 1997. Subjects whose age of onset of diabetes was ≥ 40 years and stabilized by oral antidiabetic agents and / or hygienic measures, have been identified as type 2 diabetics. Those whose age of onset was < 40 years and stabilized only by insulin therapy were identified as type 1 diabetics.

The data were collected on a survey form including demographic, clinical and biological variables. The variables studied were sex, age, type of diabetes, duration of diabetes, risk factors for HBV / C infection (history of blood transfusion, surgical operation, scarification, blood exposure accidents), blood glucose and HBV / HCV serology. The blood glucose level was determined using spectrophotometer (CYANStart 004 spectrophotometer, SN: BS1CMO 42E CYPRESS Diagnostics). The participant serologic status for HBV and HCV was determined using Determine TM^®^ HBsAg (Abbott, Tokyo, Japan) and Hexagon^®^ HCV (Human GmbH, Wiesbaden, Germany) rapid test kits respectively. Samples positive by these rapid tests were reanalyzed by ELISA (Reader 250, BIOMERIEUX, Wellwash 4 MK 2, Labsystem, Finland). The collected data were entered on Excel and analyzed by the SPSS statistics version 20.0. The arithmetic mean, standard deviation and Pearson chi-square test at the statistical significance level of 0.05 were used for the interpretation of the results.

## Results

Overall, the seroprevalence of HCV infections observed in diabetic subjects was 24.8% compared to 1.9% in volunteer blood donors with a very significant difference (p = 0.0000) (Table 1). For HBV, seroprevalence was 3.4% in diabetics compared to 3.5% among volunteer blood donors with no significant difference (p = 0.906) (Table 2). The seroprevalence of HCV by demographic, clinical and biological characteristics of diabetics is recorded in Table 3. All HCV seropositive cases were observed in subjects over 40 years of age (p = 0.002). Among type 2 diabetics, anti-HCV antibodies were found in 29.4% of subjects, compared to 6.7% in type 1 diabetics with a significant difference (p = 0.006). Subjects whose diabetes has evolved for 5 years or more had higher seropositivity than those whose disease had evolved for less than 5 years, but the difference was not significant. Among subjects with a history of blood transfusion, 50% had positive serology compared to 23% of non-transfused patients. Among those with a surgical history, 29.6% were HCV positive compared to 22.1% among those who did not have this history. However, in the latter two cases, the difference was not statistically significant. Similarly, considering the history of scarification and blood exposure accident, the study did not show a significant difference between diabetics with these antecedents and those not. Regarding HBV in diabetics, no difference in seropositivity was observed for the age, sex, type of diabetes, history of blood transfusion, surgery, scarification or blood exposure accident (p > 0.05) (Table 4).

**Table 1:** Seroprevalence of HCV in diabetic patients versus volunteer blood donors

Population	HCV+ N(%)	HCV- N(%)	Total	p value
Diabetic patients	37(24,8)	112(75,2)	149(100)	0,0000
Volunteer blood donors	101(1,9)	5158(98,1)	5259(100)	

HCV+ = HCV seropositif ; HCV- = HCV seronegatif

**Table 2:** Seroprevalence of HBV in diabetic patients versus volunteer blood donors

Population	HBV+ N (%)	HBV- N (%)	Total N (%)	p value
Diabetic patients	5(3,4)	144(96,6)	149(100)	0,906
Volunteer blood donors	186(3,5)	5073 (96,5)	5259(100)	

HBV+ = HBV seropositif; HBV- = HBV seronegatif

**Table 3:** Seroprevalence of HCV according to the demographic, clinical and biological characteristics of diabetic patients

Characteristics	Total(149)	VHC+(37)	%(24,8)	p value
**Age (year)**				0,002*
<20	4	0	0	
20-29	4	0	0	
30-39	9	0	0	
40-49	27	1	3,7	
≥50	105	36	34,3	
**Sex**				0,603
Female	63	17	27	
Male	86	20	23,3	
**Duration of diabetes millitus**				0,081
<5				
5-10	83	16	19,3	
≥10	43	16	37,2	
	23	5	21,7	
**Type of diabetes**				0,006*
Type 1	30	2	6,7	
Type 2	119	35	29,4	
**History of blood transfusion**				0,069
Yes	10	5	50	
No	139	32	23	
**History of surgical operation**				0,204
Yes	54	16	29,6	
No	95	21	22,1	
Scarification				
Yes	67	16	23,9	0,48
No	82	21	25,6	
**Blood exposure accident**				0,423
Yes	28	6	21,4	
No	121	31	25,6	

* Significant difference

**Table 4:** Seroprevalence of HBV according to the demographic, clinical and biological characteristics of diabetic patients

Characteristics	Total (149)	HBV+(5)	%(3,4)	p value
**Age (year)**				0,729
<20	4	0	0	
20-29	4	0	0	
30-39	9	0	0	
40-49	27	2	7,4	
≥50	105	3	2,9	
Sex				0,916
Female	63	2	3,2	
Male	86	3	3,5	
**Duration of diabetes mellitus**				0,892
<5	83	3	3,6	
5-9	43	1	2,3	
≥10	23	1	4,4	
Type of diabetes				0,994
Type 1	30	1	3,3	
Type 2	119	4	3,4	
**History of blood transfusion**				0,703
Yes	10	0	0	
No	139	5	3,6	
**History of surgical intervention**				0,402
Yes	54	1	1.8	
No	95	4	4.2	
**Scarification**				0,127
Yes	67	4	6	
No	82	1	1,2	
**Blood exposure accident**				0,652
Yes	28	1	3,6	
No	121	4	3,3	

## Discussion

The association between diabetes mellitus and HCV infection was reported for the first time by Allison *et al.* in 1994 and later by Simo *et al.* in 1996 [17, 18]. Since, several arguments have accumulated in favor of this association. So much has been shown that HCV infected patients have a higher risk of developing diabetes mellitus, essentially type 2 [19]. There is so much evidence that, diabetics are at high risk for HCV infection [7], but the mechanisms underlying this association remain unclear. On the other hand, concerning the seroprevalence of the HBV infection and the diabetes mellitus, controversies persist. Most studies have not observed a difference in seroprevalence of HBV between diabetic and non-diabetic subjects [7, 10, 20, 21]. Only a few studies indicate a higher risk of HBV infection in diabetics compared to non-diabetics [6, 22, 23], and a high risk of diabetes mellitus in HBV infected patients [24].

As observed in this study, the seroprevalence of HCV infection significantly higher in diabetics (24.8%) compared to volunteer blood donors (1.9%) (p = 0.0000) is in agreement with other cases reported by many authors around the world. In Korea, Ryu *et al.* (2001) reported a positive serology of 2.1% among diabetics compared to 1.3-1.6% in the general population [25]. The Ndako *et al.* study in Nigeria (2009) reported an association between diabetes mellitus and HCV infection. The prevalence found in this study was 11% whereas it was estimated at 2.1% in the general population [26]. In addition, studies in which a control group was included, found a significantly higher prevalence of anti-HCV antibodies in diabetic patients. In Ethiopia, Ali *et al.* (2012) reported that HCV seroprevalence in diabetics was 9.9% compared to 3.3% with a significant difference [27]. The same is true of Madny *et al.* study (2014) in Sudan [28] and Jadoon *et al.* (2010) in Pakistan [8], who reported that in diabetics the respective seroprevalences of HCV of 1.7% and 13.7% compared with 0% and 4.9% in the controls.

However, while agreeing with these authors, the seroprevalence of HCV infection among diabetics in the DRC (24.8%) is significantly higher than those observed elsewhere and suggests that this co-morbodity may have particularities in the DRC (related for example to HCV serotype, ethnicity), which need to be documented. Age and type of diabetes have been shown to be associated with HCV infection in diabetics. Indeed, all HCV seropositive cases were observed in subjects over 40 years, so among type 2 diabetics (Table 3). Neither the duration of diabetes mellitus nor the history of risk factors for HCV infection (blood transfusion, surgery, scarification, accidental exposure to blood) were associated with HCV infection in patients with diabetes mellitus. Similar results come from the work of Jadoon *et al.* [8] and Ali *et al.* [27]. However, despite the fact that the history of blood transfusion is not significantly associated with HCV infection in diabetics, the prevalence of this infection was high in transfused individuals (Table 3). This suggests that in the DRC, where transfusion safety coverage across the country is not yet assured, blood transfusion may be one of the factors accounting for the high seroprevalence of HCV infection in diabetics. In sub-Saharan Africa, 12.5% of transfused patients are at risk for post-transfusion hepatitis [16]. Thus, in addition to the known chronic complications of diabetes mellitus, including micro and macroangiopathies, somatic and autonomic neuropathies, the diabetic foot, an explosion of cases of hepatic cirrhosis and hepatocellular carcinoma in Congolese diabetics is to be feared in the future days if the question about the 0association viral hepatitis - diabetes mellitus is not taken seriously today.

Regarding the seroprevalence of HBV infection in diabetics (3.4%) compared with volunteer donors of blood (3.5%), no difference was observed (Table 2). Similarly, considering the history of different risk factors for HBV infection in diabetics, no difference was observed between subjects with this history and those without (Table 4). This result is similar to those of Gulcan *et al.* [7] in Turkey (2008), Mekonnen *et al.* [10] in Ethiopia (2014) who, like us, did not observe high seroprevalence of HBV in diabetics compared with non-diabetics. In addition, no risk factors for HBV infection has been associated with HBV infection in diabetics. However, a recent study conducted in the United Kingdom [23] reported a high prevalence of HBV in diabetics compared to non-diabetics and even a higher hospitalization rate in HBV-infected diabetics compared to non-infected patients. This indicates that more studies are needed in our area and systematic screening of HBV infection in diabetics is necessary.

## Conclusion

The seroprevalence of HCV and not HBV infection is significantly high in diabetic subjects, particularly type 2 diabetics, in the Democratic Republic of Congo and suggests systematic screening for these infections in any diabetic patient. More studies are needed to not only determine the factors related to this co-morbidity, but also to evaluate the impact of this infection on the evolution of diabetes mellitus and vice versa.

### What is known about this topic

- Prevalence of HCV infection in dibetic patients is high specially type 2 diabetics but results vary from one country to another;
- About the prevalence of HBV infection in diabetics, the results remain conflicting;
- In the Democratic Republic of Congo there is no report about seroprevalence of Hepatitis B and C viruses among diabetes mellitus patients.

### What this study adds

- This study provides data on the prevalence of hepatitis B and C viruses in diabetics in the Democratic Republic of Congo;
- It shows that in the Democratic Republic of Congo, the prevalence of hepatitis C infection in diabetics is higher than in other countries.

## Competing interests

The authors declare no competing interests.
